# Resilience Contributes to Low Emotional Impact of the COVID-19 Outbreak Among the General Population in Italy

**DOI:** 10.3389/fpsyg.2020.576485

**Published:** 2020-11-04

**Authors:** Vittorio Lenzo, Maria C. Quattropani, Alessandro Musetti, Corrado Zenesini, Maria Francesca Freda, Daniela Lemmo, Elena Vegni, Lidia Borghi, Giuseppe Plazzi, Gianluca Castelnuovo, Roberto Cattivelli, Emanuela Saita, Christian Franceschini

**Affiliations:** ^1^Department of Clinical and Experimental Medicine, University of Messina, Messina, Italy; ^2^Department of Humanities, Social Sciences and Cultural Industries, University of Parma, Parma, Italy; ^3^IRCCS Istituto delle Scienze Neurologiche di Bologna, Bologna, Italy; ^4^Department of Humanistic Studies, University of Naples Federico II, Naples, Italy; ^5^Department of Health Sciences, University of Milan, Milan, Italy; ^6^Department of Biomedical, Metabolic and Neural Sciences, University of Modena and Reggio Emilia, Modena, Italy; ^7^Department of Psychology, Catholic University of Milan, Milan, Italy; ^8^Istituto Auxologico Italiano IRCCS, Psychology Research Laboratory, Ospedale San Giuseppe, Verbania, Italy; ^9^Department of Medicine and Surgery, University of Parma, Parma, Italy

**Keywords:** COVID-19, clinical psychology, depression, anxiety, stress, DASS-21, resilience scale, resilience

## Abstract

**Background:**

The COVID-19 outbreak is severely affecting the overall mental health with unknown psychological consequences. Although a strong psychological impact is possible, scant evidence is available to date. Past studies have shown that resilience decreases the negative effects of stress. This study aimed to examine depression, anxiety, and stress among the Italian general population during the phase characterized by lockdown, and to investigate the role of resilience as a potential predictor.

**Methods:**

A total sample of 6,314 Italian people participated in this study. Participants were recruited between March 29 and May 04 2020 through an online survey. The Depression Anxiety Stress Scales-21 (DASS-21) and the Resilience Scale (RS) were administered. Demographic data and lockdown related information were also collected. A correlational analysis was carried out to examine relationships between psychopathological domains and resilience. Three hierarchical regression analyses were conducted using the depression, anxiety, and stress as dependent variables and the resilience as independent variable controlling for age, gender, and education. COVID-19 specific variables were also included in the three regression analyses. A further exploratory analysis was carried out to examine which aspects of resilience predict depression, anxiety, and stress.

**Results:**

The prevalence of moderate to extremely severe symptoms among participants was 32% for depression, 24.4% for anxiety, and 31.7% for stress. The sample mean scores on depression, anxiety, and stress were higher than the normal scores reported in the literature. Results of correlational analysis showed that resilience factors, such as meaningfulness, self-reliance, existential aloneness, and equanimity, are inversely associated with depression, anxiety, and stress. Results of regression analyses indicated that resilience was statically significant in predicting depression, anxiety, and stress. Geographic area of residence and infected acquaintances were also significant predictors. Regarding the resilience factors, results revealed that meaningfulness, perseverance, and equanimity were statistically significant in predicting all the DASS-21 scales.

**Conclusion:**

About a third of respondents reported moderate to extremely severe depression, anxiety, and stress. The present study suggests that psychological resilience may independently contribute to low emotional distress and psychological ill-being. These findings can help explain the variability of individual responses during the COVID-19 outbreak.

## Introduction

The coronavirus disease 2019 (COVID-19) outbreak impacted deeply on every aspect of daily life among several countries including Italy. Although the outbreak started from the Huanan Seafood Market in Wuhuan (Ahmed et al., 2020; Chen et al., 2020; Zhou et al., 2020), it has rapidly arisen with more than 5 million confirmed cases and three hundred and forty thousand deaths in the world (WHO report). Since the World Health Organization (World Health Organization, 2020) declared the COVID-19 outbreak a pandemic on March 11, many countries adopted restrictive measures never seen before. A massive lockdown was implemented by the Italian Government to decelerate the spread of the virus. This was an unprecedented decision concerning more than 60 million people in total with an unknown psychological impact. Most of the interventions implemented by health care authorities have focused on physical health including medical therapies and paid less attention to the psychological impact of the outbreak and the resulting lockdown. In the past, the containment measures following a severe outbreak were imposed in limited areas, such as for the 2003 outbreak of severe acute syndrome (SARS) even though with some similarities. A recent review including 24 studies found negative psychological effects among the population affected by the lockdown including post-traumatic symptoms, confusion, and anger (Brooks et al., 2020). However, the generalizability of these findings is still limited because of not specifically referring to the COVID-19 outbreak. Although evidence on the psychological impact of the COVID-19 remains unknown, early studies have provided some important results. A study among a large sample of Chinese people has found that about a third of the 52,730 participants reported psychological distress (Qiu et al., 2020). Also, in predicting psychological distress, the following risk factors have been identified: female gender, young or elderly age, and higher education. Another study involving a sample of 1210 participants from several Chinese cities reported a prevalence of severe depression, anxiety, and stress ranging from 8 to 29%, with most of the respondents considering the psychological impact of outbreak as moderate or severe (Wang et al., 2020a). Moreover, no significant decrease in negative psychological effects was detected among a 4-week period (Wang et al., 2020b). Similar results were found by an epidemiological study on a sample of 2,812 Italian participants with a prevalence of severe psychological symptoms of 32.8% for depression, 18.7% for anxiety, and 27.2% for stress (Mazza et al., 2020). Similar results were found by another study among Italian people during the initial phase of outbreak. Moccia et al. (2020) reported a prevalence of mild and moderate-to-severe psychological distress of almost 20 percent. A central tenet of this study was to examine the role of attachment in predicting psychological distress following the COVID-19 outbreak. In this perspective, findings pointed out that insecure attachment dimensions would be considered as risk factors for moderate-to-severe distress. Previous studies have demonstrated the relationships between psychological functioning and psychological distress among a wide array of populations (Bowlin and Baer, 2012; Lenzo et al., 2020a,b). From this perspective, there is growing evidence of resilience as a protective factor against negative psychological effects. In the last years, a growing number of researchers have confirmed the role that resilience has in the adjustment to adversity (deRoon-Cassini et al., 2010; Southwick et al., 2014; MacLeod et al., 2016; Schäfer et al., 2018; Van der Meer et al., 2018). From this perspective, resilience has been identified as a central target and it is worthwhile to enhance it among people during the COVID-19 outbreak (Khan et al., 2020). Although resilience is a multifaceted construct, a well-consolidated research framework explains resilience as a personality feature that mitigates the negative consequences of stress (Wagnild and Young, 1993). Meaningfulness, self-reliance, perseverance, existential aloneness, and equanimity are the five components underlying resilience (Wagnild and Young, 1990). Previous studies have found that resilience is inversely associated with poor mental health and depression and positively with meaning in life and self-efficacy (Girtler et al., 2010; Damásio et al., 2011; Surzykiewicz et al., 2019). To date, little is known about the relationships between psychological resilience and distress during the COVID-19 outbreak. This is surprising because a considerable amount of research has well demonstrated how resilience is inversely related to the impact of adversity, threats, or relevant sources of stress.

The first aim of this study was to examine the prevalence of depression, anxiety, and stress among a large sample of Italian people. We hypothesized about one-third of the prevalence rate for moderate to severe psychological distress and higher scores than the normal range. The second aim of this study was to explore the relationships between resilience and depression, anxiety, and stress. We hypothesized that we would find inverse relationships between resilience and depression, anxiety, and stress. Finally, the third aim of this study was to investigate the role of resilience in explaining depression, anxiety, and stress. We hypothesized that resilience would significantly relate to psychological symptoms.

## Materials and Methods

### Participants and Procedure

A cross-sectional design to assess psychological response during the COVID-19 outbreak in Italy was adopted. Data presented in this study are part of a larger and multicentre research project named “Resilience and the COVID-19: how to react to perceived stress. Effects on sleep quality and diurnal behavior/thoughts.” A total of 6,314 subjects participated in this study through an online survey system without any form of compensation. Thirty-seven cases were excluded for incomplete data and 622 were identified as outliers and removed from the sample. Consequently, the final sample consisted of 5,655 subjects, as shown in the Table 1. Participants ranged in age from 18 to 81 (*M* = 33.63, *SD* = 13.40) and 72.2% of the sample were female (*n* = 4082). Most of the participants were living in Northern Italy (68.5%). Less than half of the sample had a high school diploma (46.3%) and 93.6% (*n* = 5313) were employed or students. With regard to marital status, 39.3% were unmarried, divorced, or widowed. More than half of the sample worked during the lockdown (*n* = 3057, 54.1%), mostly as remote work (*n* = 1840, 60.2%). Eight percent (*n* = 470) of the respondents were in mandatory quarantine for COVID-19. A proportion of 14.9% (*n* = 840) declared that at least a loved one had been infected by COVID-19.

**TABLE 1:** Demographic characteristics of the sample.

**Variable**	**Mean**	***SD***	***n***	**Percentage**
Age (in years)	33.63	13.40		
**Gender**				
Male			1573	27.8%
Female			4082	72.2%
**Education**				
Primary or middle school diploma			180	3.2%
High school diploma			2621	46.3%
Graduate			2378	42.0%
Postgraduate			476	8.4%
**Partnership**				
Unmarried, divorced or widowed			2222	39.3%
Married or in a steady partnership			3423	60.7%
**Having children**				
Yes			1625	28.7%
No			4030	71.3%
**Area of residence**				
Northern Italy			3871	68.5%
Central-southern Italy			1784	31.5%
**Work status**				
Employed or student			5313	93.95%
Unemployed or retired			342	6.05%
**Work during the lockdown**				
No			2598	45.9%
Yes			3057	54.1%
**Work modality (*n* = 3057)**				
Exclusively in-person work			623	20.4%
Exclusively remote work			1840	60.2%
Mixed (remote and in-person work)			224	7.3%
Mostly in-person work			103	3.4%
Mostly remote work			239	7.8%
No answer			28	0.9%
**Number of household members**				
≤2			3021	53.4%
3			1691	29.9%
4			715	12.6%
≥5			228	4.1%
**Mandatory quarantine for COVID-19**				
No			5166	91.4%
Yes			470	8.3%
No answer			19	0.3%
**Infected acquaintances or loved ones**				
No			4815	85.1%
Yes			840	14.9%
**Death of loved ones for COVID-19**				
No			5295	93.7%
Yes			358	6.3%

*N = 5655*

### Ethical Statement

The current study was conducted in accordance with the 1964 Declaration of Helsinki and its later amendments. The study was approved by the Research Ethics Committee for Psychological Research of the University of Messina, Italy (n. 37442). The participants provided their written informed consent to participate in this study.

### Measures

#### Socio-Demographics

Socio-demographic variables included age, gender, education, relationship status, employment status, and residential location during the COVID-19 outbreak. Also, additional information related to COVID-19 was collected (i.e., if loved ones had been infected, family composition, etc.). Table 1 reports the demographic characteristics of the sample.

#### Depression, Anxiety, and Stress

The Depression Anxiety Stress Scale – 21 (DASS-21) (Lovibond and Lovibond, 1995) was used to measure depression, anxiety, and stress. The DASS-21 is a 21-item self-report instrument using a four-point Likert scale ranging from “never” (0) to “always” (3). It consisted of the following three scales: depression (e.g., “I couldn’t seem to experience any positive feeling at all”), assessing dysphoria, low self-esteem, anhedonia, lack of interest, and passivity; anxiety (e.g., “I was aware of dryness of my mouth”), comprising somatic and subjective symptoms of anxiety; stress (e.g., “I found it hard to wind down”), evaluating persistent arousal, irritability, psychological tension, and agitation. In the current study, the Italian version of DASS-21 showing excellent psychometric properties was adopted (Bottesi et al., 2015). Excellent levels of reliability were detected in this sample (Depression, α = 0.89; Anxiety, α = 0.83; Stress, α = 0.90).

#### Resilience

The Wagnild and Young Resilience Scale (RS) (Wagnild and Young, 1993) was used to measure resilience which is defined as a personal and positive characteristic that enhances individual adaptation to adversity. The Italian version of RS is a 24-item self-report instrument (e.g., “When I make plans I follow through with them”) using a 7-point Likert scale to “1” (*disagree*) to “7” (*agree*) (Girtler et al., 2010). The items are grouped into five scales as follows: Meaningfulness (e.g., “My life has meaning”), which measures the sense of having something for which live; Self-reliance (e.g., “When I am in a difficult situation, I can usually find my way out of it”), which measures the beliefs in oneself and one’s abilities; Perseverance (e.g., “Sometimes I make myself do things whether I want to or not”), which measures perseverance despite adversity or discouragement; Existential aloneness (e.g., “I am able to depend on myself more than anyone else”), which measures feeling of freedom and sense of uniqueness; and Equanimity (e.g., “I do not dwell on things that I can’t do anything about”), which measures a balanced perspective vision of one’s life and experience. Also, it is possible to obtain a total score of the RS with higher scores indicating high resilience. Specifically, values of 126.6 and above indicate high resilience (Girtler et al., 2010). Previous studies have shown that the RS is a reliable and sample tool with good psychometric properties (Wagnild and Young, 1993; Aroian et al., 1997; Heilemann et al., 2003; Lundman et al., 2007; Girtler et al., 2010). In this study, the degree of reliability of the five scales was from acceptable to good, with a Cronbach’s α of 0.65 for self-reliance, 0.71 for perseverance, 0.78 for equanimity, 0.80 for existential aloneness, 0.89 for meaningfulness, and 0.94 for the total score.

### Statistical Analysis

Statistical analysis was performed using IBM SPSS Statistics version 22 (IBM Corporation, Armonk, NY, United States). Data obtained from this study were checked to detect and remove outliers and incomplete data (Tabachnick and Fidell, 2013). Subsequently, descriptive and inferential statistical analyses were conducted. Relationships between RS and DASS-21 were performed with Pearson product-moment correlation coefficients. To examine the relationship between depression, anxiety, and stress with resilience, three hierarchical regression analyses were conducted, each one including three steps. Depression, anxiety, and stress were set as dependent variables. Age, gender, and education were inserted as covariates in all the three steps. In the second step, we inserted COVID-19 specific variables as follows: geographic area of residence, mandatory quarantine, infected acquaintances or loved ones, and the death of loved ones due to COVID-19. Since the mandatory quarantine included the possibility that the respondents did not answer (i.e., “I prefer not to answer”), we excluded from the regression analyses all these cases. Lastly, we inserted the resilience total score in the regression analyses. Additionally, we carried out the three hierarchical regression analyses with the resilience factors (i.e., meaningfulness, self-reliance, perseverance, existential aloneness, and equanimity) in the third step to explore which aspects of resilience are related to the dependent variables.

## Results

### Prevalence of Depression, Anxiety, and Stress and Relationships With the Response Time

Table 2 displays the percentage of participants falling into each of the five categories, such as normal, mild, moderate, severe, and extremely severe based on the Lovibond and Lovibond’s percentile cut-offs (1995). The overall prevalence of moderate-to-extremely severe depression, anxiety, and stress among participants was 32, 24.4, and 31.7%, respectively. The last column of Table 2 reports correlation coefficients between the DASS-21 scales and the response time from the lockdown start. Depression and stress scales showed a weak and positive correlation coefficient (respectively, *r* = 0.03, *p* < 0.05 and *r* = 0.04, *p* < 0.01) with time since the lockdown start. Table 3 presents the descriptive statistics for the three DASS-21 scales. The mean score for depression, anxiety, and stress was 10.33 (*SD* = 8.21), 5.75 (*SD* = 5.73), and 14.81 (*SD* = 9.04), respectively.

**TABLE 2:** Prevalence of depression, anxiety and stress.

	**Percentage in each DASS category**					**Response time**
	**Normal (0–78)**	**Mild (78–87)**	**Moderate (87–95)**	**Severe (95–98)**	**Extremely severe (98–100)**	***r***
Depression	51.5	16.6	19.2	8.1	4.7	0.03*
Anxiety	67.9	7.7	14.6	6.2	3.6	0.02
Stress	55.3	13.0	17.0	11.6	3.1	0.04**

N = 5655. The percentiles in parentheses corresponding to Lovibond and Lovibond’s cut-offs (1995). *p < 0.05, **p < 0.01.

**TABLE 3:** Descriptive and correlational analyses.

	**Min**	**Max**	***M***	***SD***	**Skew**	**Kurt**	**(1) RS meaning fulness**	**(2) RS self- reliance**	**(3) RS perseverance**	**(4) RS existential aloneness**	**(5) RS equanimity**	**(6) RS total score**	**(7) DASS-21 depression**	**(8) DASS-21 anxiety**
(1) RS meaningfulness	13.00	49.00	37.67	6.93	–0.60	–0.04								
(2) RS self-reliance	12.00	45.00	33.06	5.83	–0.39	–0.29	0.69**							
(3) RS perseverance	3.00	21.00	15.53	3.15	–0.55	0.03	0.74**	0.66**						
(4) RS existential aloneness	5.00	21.00	17.50	2.78	–1.04	1.09	0.67**	0.56**	0.56**					
(5) RS equanimity	3.00	21.00	16.49	3.27	–0.78	0.29	0.68**	0.58**	0.63**	0.60**				
(6) RS total score	70.00	171.00	131.23	19.93	–0.59	0.01	0.92**	0.84**	0.83**	0.77**	0.80**			
(7) DASS-21 depression	0	34.00	10.33	8.21	0.80	–0.06	−0.36**	−0.27**	−0.39**	−0.26**	−0.34**	−0.38**		
(8) DASS-21 anxiety	0	22.00	5.75	5.73	1.05	0.25	−0.21**	−0.15**	−0.23**	−0.14**	−0.22**	−0.22**	0.55**	
(9) DAS-21 stress	0	42.00	14.81	9.04	0.36	–0.45	−0.27**	−0.20**	−0.30**	−0.14**	−0.25**	−0.27**	0.72**	0.63**

N = 5655. Min, minimum value; Max, maximum value; M, mean; SD, standard deviation; Skew, skewness; Kurt, kurtosis. **p < 0.01.

### Correlational Analysis Between Resilience, Depression, Anxiety, and Stress

Table 3 shows descriptive statistics and correlation analyses. Results showed that all the RS scales were all positively and highly correlated with each other, and with the RS total core. Likewise, depression, anxiety, and stress scales were positively correlated with each other. Also, correlational analyses showed that meaningfulness, self-reliance, perseverance, existential aloneness, equanimity, as well as the RS total score were weakly and negatively correlated with depression, anxiety, and stress.

### Regression Analyses for Depression, Anxiety, and Stress

Table 4 shows the regression results of the effects of resilience and COVID-19 specific variables controlling for age, gender, and level of education on depression, anxiety, and stress. In predicting depression, age (β = −0.19; *p* < 0.001), gender (β = −0.10; *p* < 0.001), and education (β = −0.02; *p* < 0.001) were all statistically significant in step 1. In step 2, the effect of age (β = −0.18; *p* < 0.001) and gender (β = −0.10; *p* < 0.001) persisted. In addition, area of residence (β = 0.03; *p* = 0.039) and infected acquaintances or loved ones (β = 0.05; *p* < 0.001) were statistically significant to explain depression levels. The results showed the same effects on depression in step 3, as illustrated in the Table 4. Furthermore, we observed a statistically significant effect of resilience on depression (β = −0.36; *p* < 0.001) with *R*^2^ reaching 0.18. The second regression analyses tested the same model although considering anxiety as the dependent variable. In step 1, age (β = −0.11; *p* < 0.001), gender (β = −0.16; *p* < 0.001), and education (β = −0.04; *p* < 0.001) were all statistically significant. In step 2, after adding the COVID-19 variables, age (β = −0.10; *p* < 0.001), gender (β = −0.15; *p* < 0.001), and education (β = −0.05; *p* < 0.001) maintained a significant effect. In addition, area of residence (β = 0.06; *p* < 0.001) and infected acquaintances or loved ones (β = 0.05; *p* < 0.001) reached a statistical significance. We found the same effects of these factors in step 3. Moreover, the resilience score inserting in step 3 was statistically significant (β = −0.20; *p* < 0.001) with *R*^2^ of the model reaching 0.09. Lastly, the third regression analyses examined stress as the dependent variable. In step 1, age (β = −0.23; *p* < 0.001), gender (β = −0.15; *p* < 0.001), and education (β = 0.04; *p* = 0.003) were statistically significant. In step 2, we observed the same effects for age (β = −0.23; *p* < 0.001), gender (β = −0.15; *p* < 0.001), and education (β = 0.04; *p* = 0.004). Furthermore, infected acquaintances or loved ones (β = 0.06; *p* < 0.001) was a statistically significant factor in predicting stress. In step 3, lastly, these effects persisted and we also observed a significant effect for area of residence (β = 0.03; *p* = 0.042), mandatory quarantine for COVID-19 (β = 0.03; *p* = 0.036), and resilience score (β = −0.25; *p* < 0.001).

**TABLE 4:** The regression results of the effects of sociodemographic variables and resilience on depression, anxiety and stress.

	**Partial regression coefficient**				***R*^2^**	***F***	***p***
	**β**	***SE***	***T***	***p***			
**Depression**							
*Step 1*					0.05	97.96	<0.001
Age	–0.19	0.01	–13.82	<0.001			
Gender	–0.10	0.24	–7.79	<0.001			
Level of education	–0.02	0.11	–1.58	<0.001			
*Step 2*					0.05	44.55	<0.001
Age	–0.18	0.01	–13.53	<0.001			
Gender	–0.10	0.24	–7.63	<0.001			
Education	–0.03	0.10	–1.88	0.061			
Area of residence	0.03	0.24	2.06	0.039			
Mandatory quarantine for COVID-19	0.01	0.39	0.32	0.746			
Infected acquaintances or loved ones	0.05	0.32	3.25	<0.001			
Death of loved ones for COVID-19	0.02	0.45	3.25	0.232			
*Step 3*					0.18	153.99	<0.001
Age	–0.14	0.01	–11.30	<0.001			
Gender	–0.09	0.22	–7.30	<0.001			
Level of education	–0.12	0.10	–1.00	0.319			
Area of residence	0.05	0.22	4.08	<0.001			
Mandatory quarantine for COVID-19	0.01	0.36	0.53	0.593			
Infected acquaintances or loved ones	0.03	0.29	2.51	0.012			
Death of loved ones for COVID-19	0.02	0.42	1.32	0.187			
RS total score	–0.36	0.01	–29.53	<0.001			
**Anxiety**							
*Step 1*					0.04	89.94	<0.001
Age	–0.11	0.01	–8.22	<0.001			
Gender	–0.16	0.17	–11.92	<0.001			
Education	–0.04	0.07	–3.13	<0.001			
*Step 2*					0.05	42.68	<0.001
Age	–0.10	0.01	–7.66	<0.001			
Gender	–0.15	0.17	–11.67	<0.001			
Education	–0.05	0.07	–3.61	<0.001			
Area of residence	0.06	0.16	4.18	<0.001			
Mandatory quarantine for COVID-19	0.04	0.27	3.22	<0.001			
Infected acquaintances or loved ones	0.05	0.22	3.72	<0.001			
Death of loved ones for COVID-19	0.02	0.32	1.30	0.192			
*Step 3*					0.09	69.27	<0.001
Age	–0.08	0.01	–6.11	<0.001			
Gender	–0.15	0.16	–11.44	<0.001			
Level of education	–0.04	0.07	–3.15	<0.001			
Area of residence	0.07	0.16	5.25	<0.001			
Mandatory quarantine for COVID-19	0.04	0.27	3.38	<0.001			
Infected acquaintances or loved ones	0.04	0.22	3.28	<0.001			
Death of loved ones for COVID-19	0.02	0.31	1.35	0.177			
RS total score	–0.20	0.01	–15.58	<0.001			
**Stress**							
*Step 1*					0.08	160.92	<0.001
Age	–0.23	0.01	–17.65	<0.001			
Gender	–0.15	0.26	–11.40	<0.001			
Education	0.04	0.11	3.01	0.003			
*Step 2*					0.08	73.27	<0.001
Age	–0.23	0.01	–17.17	<0.001			
Gender	–0.15	0.26	–11.32	<0.001			
Education	0.04	0.11	2.86	0.004			
Area of residence	0.01	0.26	0.76	0.448			
Mandatory quarantine for COVID-19	0.03	0.42	1.91	0.056			
Infected acquaintances or loved ones	0.06	0.34	4.08	<0.001			
Death of loved ones for COVID-19	0.02	0.49	1.22	0.224			
*Step 3*					0.14	116.96	<0.001
Age	–0.20	0.01	–15.55	<0.001			
Gender	–0.14	0.25	–11.10	<0.001			
Level of education	0.05	0.11	3.64	<0.001			
Area of residence	0.03	0.25	2.03	0.042			
Mandatory quarantine for COVID-19	0.03	0.41	2.10	0.036			
Infected acquaintances or loved ones	0.05	0.33	3.56	<0.001			
Death of loved ones for COVID-19	0.02	0.48	1.28	0.200			
RS total score	–0.25	0.01	–19.69	<0.001			

*n = 5636. RS, Resilience Scale.*

Table 5 illustrates the regression results of the effects of demographic variables and resilience factors on depression, anxiety and stress. Regarding demographic variables, we found analogous results to the last ones considering the RS total score. On the other hand, resilience factors are specifically related to the DASS-21 scales. Meaningfulness (β = −0.09; *p* < 0.001), self-reliance (β = 0.04; *p* = 0.038), perseverance (β = −0.24; *p* < 0.001), and equanimity (β = −0.15; *p* < 0.001) factors were all statistically significant to explain depression. Meaningfulness (β = −0.06; *p* = 0.010), self-reliance (β = 0.04; *p* = 0.027), perseverance (β = −0.14; *p* < 0.001), and equanimity (β = −0.12; *p* < 0.001) factors were also significant in predicting anxiety. Lastly, meaningfulness (β = −0.12; *p* < 0.001), perseverance (β = −0.19; *p* < 0.001), existential aloneness (β = −0.11; *p* < 0.001), and equanimity (β = −0.12; *p* < 0.001) factors were statistically significant in predicting stress. Differently from depression and anxiety models, existential aloneness but not self-reliance was statistically significant.

**TABLE 5:** The regression results of the effects of demographic variables and resilience factors on depression, anxiety and stress.

	**Partial regression coefficient**				***R*^2^**	***F***	***p***
	***β***	***SE***	***t***	***P***			
Depression*							
*Step 3*					0.20	120.41	<0.001
Age	–0.14	–0.14	–11.13	<0.001			
Gender	–0.09	–0.09	–7.07	<0.001			
Level of education	–0.12	0.01	0.07	0.946			
Area of residence	0.05	0.22	3.80	<0.001			
Mandatory quarantine for COVID-19	0.01	0.36	0.31	0.755			
Infected acquaintances or loved ones	0.03	0.29	2.34	0.019			
Death of loved ones for COVID-19	0.02	0.42	1.36	0.175			
RS meaningfulness	–0.09	0.03	–4.11	<0.001			
RS self-reliance	0.04	0.03	2.08	0.038			
RS perseverance	–0.24	0.05	–12.64	<0.001			
RS existential aloneness	0.01	0.05	0.48	0.635			
RS equanimity	–0.15	0.04	–8.27	<0.001			
Anxiety*							
*Step 3*					0.10	54.55	<0.001
Age	–0.08	0.01	–5.96	<0.001			
Gender	–0.14	0.16	–11.21	<0.001			
Level of education	–0.03	0.07	–2.43	<0.001			
Area of residence	0.07	0.16	5.18	<0.001			
Mandatory quarantine for COVID-19	0.04	0.27	3.30	<0.001			
Infected acquaintances or loved ones	0.04	0.21	3.16	0.002			
Death of loved ones for COVID-19	0.02	0.31	1.36	0.174			
RS meaningfulness	–0.06	0.02	–2.58	0.010			
RS self-reliance	0.04	0.02	2.21	0.027			
RS perseverance	–0.14	0.04	–6.75	<0.001			
RS existential aloneness	0.03	0.04	1.66	0.097			
RS equanimity	–0.12	0.03	–6.45	<0.001			
Stress*							
*Step 3*					0.17	96.07	<0.001
Age	–0.19	0.01	–15.08	<0.001			
Gender	–0.13	0.25	–10.77	<0.001			
Level of education	0.05	0.11	4.08	<0.001			
Area of residence	0.03	0.24	2.15	0.031			
Mandatory quarantine for COVID-19	0.02	0.40	1.97	0.049			
Infected acquaintances or loved ones	0.04	0.33	3.46	<0.001			
Death of loved ones for COVID-19	0.02	0.47	1.15	0.249			
RS meaningfulness	–0.12	0.03	–5.22	<0.001			
RS self-reliance	0.03	0.03	1.39	0.164			
RS perseverance	–0.19	0.06	–9.80	<0.001			
RS existential aloneness	–0.11	0.06	6.60	<0.001			
RS equanimity	–0.12	0.05	–6.61	<0.001			

n = 5636. *We did not report the step 1 and 2 of the regression analyses. RS, Resilience Scale.

## Discussion

### Summary of the Main Findings

During the most critical weeks of the COVID-19 outbreak, Italy’s government adopted a massive lockdown to prevent the spread of the virus. This study aimed to examine mental health in a large sample of Italian people and to investigate the role of resilience as a protective factor for negative psychopathological consequences. Evidence from restrictive measures and isolation relating to past outbreaks highlighted a high risk for developing mental health disorders with possible long-lasting effects (Brooks et al., 2020; Roychowdhury, 2020). However, the COVID-19 outbreak has for the first time concerned worldwide and severely involved Italy. Although health authorities launched a psychological online service, little is known about the psychological impact of the COVID-19. Our findings pointed out a high prevalence with about one-third of participants reported moderate to extremely severe symptoms of depression, anxiety, and stress. Results of descriptive statistics suggested a possible relevant psychological impact of the COVID-19 outbreak. Participants of this study had higher levels of depression, anxiety, and stress than the normal range reported by the Italian validation of the DASS-21 (Bottesi et al., 2015). Although some differences in the sample composition, it is reasonable to think that the values reported in this study are unusual and particularly higher than the normal range of prevalence. A recent study conducted during the Italian lockdown by Mazza et al. (2020) reported similar results even if with slightly lower scores. This result could be in part dependent on the period of the survey as Mazza et al. (2020) referred to immediate psychological responses since they collected the data from 18 to 22 March. Differently, our survey started later, towards the end of March, when Italy had been on lockdown for more weeks. In light of this perspective, it could be reasonable to hypothesize an incremental rate of psychopathological symptoms over time. Although we have taken into account this hypothesis, our results indicate only a small correlation of depression and stress with the time elapsed from the start of the lockdown. It is worthwhile to consider that no significant relationship was found between anxiety and response time. We state that these relationships could be mediated by several factors, such as individual characteristics, suggesting the lack of a direct effect of time response on depression, anxiety, and stress. Longitudinal data are needed to verify these hypotheses, even if preliminary data have confirmed that there were no changes in depression, anxiety, and stress levels in a 4-week period (Wang et al., 2020b). Studies involving Chinese people have found results in part different with the prevalence rate of psychological complaints ranging from 8 to 29% (Wang et al., 2020a,b).

The second aim of this study was to examine the relationship between depression, anxiety, stress, and resilience. Previous studies demonstrated that psychological resilience promotes mental health and adaption in the face of traumatic experiences or adverse events (Southwick et al., 2014). According to Wagnild and Young (1993), resilience is a multicomponent construct comprising the sense of having something for which live, the beliefs in oneself, the perseverance degree in the face of adversity, personal feelings of freedom and distinctiveness, and a stable perspective of one’s life. It is logical to assume that these aspects have been proven during the most critical weeks of the lockdown. For this reason, we hypothesized inverse relationships between psychological symptoms and resilience. In line with the hypothesis, the resilience dimension was negatively correlated with depression, anxiety, and stress. We obtained similar results when adopting the resilience factors, even though we considered these results as exploratory. The results from the correlational analysis indeed confirmed the inverse relationships between the resilience factors and depression, anxiety, and stress. Past studies have well established these relationships (Girtler et al., 2010; Damásio et al., 2011; Surzykiewicz et al., 2019), even though our results specifically referred to people during the quarantine. While studies regarding the psychological impact of the COVID-19 have focused on the prevalence of psychological distress (Qiu et al., 2020; Wang et al., 2020a), none have explored relationships with resilience.

The third aim of this study was to examine the role of resilience dimensions in predicting depression, anxiety, and stress among a large sample of Italian people during the lockdown. Demographic data were included in the regression analysis given their contribution in predicting post-traumatic stress symptoms, depression, anxiety, and stress during the early stages of the COVID-19 outbreak (Qiu et al., 2020; Wang et al., 2020a,b). The results showed a significant effect of gender and age on depression. Also, gender, age, and education were statistically significant in predicting anxiety as well as stress. This is not surprising when we take into account findings from the literature on community samples. Previous studies have found that females had higher scores than males on depression, anxiety, and stress (Crawford and Henry, 2003; Norton, 2007), even though there was no consistency across the studies (Bottesi et al., 2015). However, it is worthwhile to highlight that a significant role of gender in predicting distress during the COVID-19 outbreak was found across countries, including Italy (Mazza et al., 2020; Wang et al., 2020a). Analogous issues have been described in the literature when considering age and education. Referring to past outbreaks, a worse psychological impact was associated with younger age and lower level of education (Brooks et al., 2020). Our findings seem to confirm the role of such demographic variables in explaining psychological impact during the lockdown following the COVID-19 outbreak. However, with regard to the COVID-19 outbreak, their results are only in part confirmed and more research is needed. For example, age was found to be related to higher stress but not to depression and anxiety (Mazza et al., 2020), even though referring to an early period of lockdown. It is reasonable to hypothesize that the lack of consistent results represents a preexisting critical issue (Bottesi et al., 2015) depending on several factors such as the sample composition. Regarding the role of information specific to COVID-19, we found more coherence when comparing with literature focusing on the COVID-19 outbreak in Italy. In this light, having an acquaintance or loved one infected with COVID-19 was associated with higher levels of depression, anxiety, and stress. Analogous results were detected by Mazza et al. (2020). Conversely, (Wang et al., 2020a) found no significant effect among their sample of participants in China. Surprisingly, we found that being in mandatory quarantine was related to anxiety and stress but not to depression. This could be in part depend by the overlap between anxiety and stress (Bottesi et al., 2015). The results also showed a significant effect for the area of residence, with participants who lived in Northern Italy scoring significantly higher than others on depression, anxiety, and stress. Findings from another recent study pointed out a higher prevalence of anxiety in the Lombardy region than the rest of Italy (Chirico et al., 2020). Nonetheless, these findings should be taken with caution since the possible and unavoidable imbalance of some COVID-19 information. Although the relevance of these findings focusing on demographic features predicting psychological distress, our main aim was to examine the specific contribution of psychological resilience. Understanding the psychological factors associated with distress among people during the COVID-19 outbreak is necessary to construct evidence-based interventions. Coherent with we expected, resilience was related to depression, anxiety, and stress. We also investigated which resilience factors are associated with psychological distress among the respondents. Although these findings can be considered only exploratory, we believe they can enhance our comprehension of the resilience role. Meaningfulness, self-reliance, perseverance, and equanimity were significant predictors of both depression and anxiety among Italian people during the COVID-19 outbreak. We found analogous results for stress except for self-reliance in the last regression analysis. On the other hand, existential aloneness was related to stress but not to depression and anxiety. Overall, these results suggest that the resilience components play a relevant role to explain distress. Nonetheless, the contribution of demographic data should be careful to take into account. The high prevalence rate of psychological symptoms founding among Chinese and Italian people involved in the lockdown has highlighted the need to consider mental health together with the fight of COVID-19 disease. Examining the role that psychological factors have for the development and maintenance of depression, anxiety, and stress is fundamental to detect people at risk of psychological disorders and to design evidence-based interventions (Castelnuovo et al., 2020). From this perspective, Moccia et al. (2020) have provided first evidence on the role of temperament and attachment style dimensions in predicting the psychological impact of the COVID-19 outbreak. More research is needed to confirm these findings and to verify the long-lasting effects of individual differences in the mental health of people who experienced the lockdown related to the COVID-19 outbreak.

### Limitations

The current study has some limitations that should be addressed by future research and considered in understanding the results. First, this study adopted a cross-sectional design that did not allow establishing causal relationships between the observed variables. Longitudinal studies would better explain the long-lasting impact of resilience dimensions on depression, anxiety, and stress development among people who experienced the COVID-19 outbreak. This research is currently underway by the authors. Second, this study involved convenience sample recruitment that could have limited the generalizability of the results. The oversampling of some characteristics among the respondents (i.e., gender or work status) could influence the results obtained. Despite the possible selection bias related to our sample, our choice was the only solution to collect the data during the lockdown. The third limitation concerns the use of self-assessment instruments to measure depression, anxiety, and stress levels. Although the DASS-21 is a reliable and widely used tool, social desirability could affect results.

### Conclusion

The negative psychological impact of restrictive measures following an outbreak is well documented. Nonetheless, there is still a paucity of studies focused on the COVID-19 outbreak. The results of our study pointed out that about a third of people reported moderate to extremely severe symptoms of depression, anxiety, and stress during the COVID-19 lockdown. Differences in the experienced severity of these symptoms seem to in part dependent on resilience dimensions. To our knowledge, this is the first attempt to examine the relationships between resilience and psychological symptoms among a large sample of Italian people. Starting from these results, psychological interventions focused on resilience could be useful to decrease the psychological impact of quarantine measures. Nonetheless, some limitations such as the cross-sectional design should be addressed by future research to clarify the role of resilience over time.

## Data Availability Statement

The raw data supporting the conclusions of this article will be made available by the authors, without undue reservation.

## Ethics Statement

The studies involving human participants were reviewed and approved by Research Ethics Committee for Psychological Research of the University of Messina, Italy. The patients/participants provided their written informed consent to participate in this study.

## Author Contributions

CF provided substantial contributions to the conception of the work, deep analysis of the literature, study design, development, and final approval of the manuscript. AM contributed in the design of the study and participated in the development and revision of the work and agreement for final approval of the manuscript. VL contributed to data analysis, to write the first draft and agreement for final approval of the manuscript. CZ, MF, DL, EV, LB, GP, GC, and RC contributed to the revision of the work and agreement for final approval of the manuscript. MQ and ES contributed to deep revision of the work, with literature analysis and agreement for final approval of the manuscript. All authors contributed to the article and approved the submitted version.

## Conflict of Interest

GP participated in advisory board for UCB Pharma, Jazz Pharmaceuticals, and Bioproject. The remaining authors declare that the research was conducted in the absence of any commercial or financial relationships that could be construed as a potential conflict of interest. The handling editor is currently organizing a Research Topic with one of the authors GC.
